# The value of mastectomy flap fixation in reducing fluid drainage and seroma formation in breast cancer patients

**DOI:** 10.1186/1477-7819-10-8

**Published:** 2012-01-11

**Authors:** Mostafa A Sakkary

**Affiliations:** 1 Surgery department, National Cancer Institute (NCI), Kasr El-Aini St., Fom El-Khalig, Cairo 11796, Egypt

**Keywords:** mastectomy, flap, fixation, seroma

## Abstract

**Background:**

Prolonged and excessive drainage of serous fluid and seroma formation constitute the most common complications after mastectomy for breast carcinoma. Seroma formation delays wound healing, increases susceptibility to infection, skin flap necrosis, persistent pain and prolongs convalescence. For this, several techniques have been investigated to improve primary healing and minimize seroma formation.

**Materials and methods:**

Between June 2009 and July 2010 forty patients with breast carcinoma, scheduled for modified radical mastectomy, were randomly divided into 2 groups, the study group (20) and the control group (20). In the study group; the mastectomy flaps were fixed to the underlying muscles in raws, at various parts of the flap and at the wound edge using fine absorbable sutures. In the control group; the wound was closed in the conventional method at the edges. Closed suction drains were used in both groups. Patients, tumor characteristics and operative related factors were recorded. The amount and color of drained fluid were recorded daily. The drains were removed when the amount become less than 50 cc. The total amount and duration of drained fluid and the formation of seroma were recorded and the results were compared between the two groups.

**Results:**

In the flap fixation group, the drain was removed in significantly shorter time compared to the control group (p < 0.001). Also, the total amount of fluid drained was significantly lower in the flap fixation group (p < 0.001). The flap fixation group showed a significantly lower frequency of seroma formation compared to the control group, both clinically (p = 0.028) and ultrasonographically (p = 0.047).

**Conclusions:**

The mastectomy flap fixation technique is a valuable procedure that significantly decreases the incidence of seroma formation, and reduces the duration and amount of drained fluid. However, it should be tried on a much wider scale to prove its validity.

## Background

Seroma and prolonged, excessive drainage of serous fluid constitute the most common complications after mastectomy for breast carcinoma. The reported incidence of seroma formation varies between 15 and 81% [1-4].

Seroma formation increases the risk of post operative complications; delays wound healing, increases susceptibility to infection, skin flap necrosis, persistent pain and wound dehiscence and prolongs convalescence [5]. The Egyptian females are characterized by having large sized breasts, which cause prolonged excessive drainage of fluids after mastectomy due to large raw areas.

Ideal wound closure should minimize lymph spillage and serum oozing, provide a means of holding skin flaps securely to the chest wall structures, obliterate dead space, and allow rapid removal of fluid as it forms. For this, several techniques of flap fixation or wound drainage, as well as limitation of postoperative shoulder movement and the use of adhesive glue, have been investigated to improve primary healing and minimize seroma formation [6,7].

The aim of the this study is to evaluate the effect of obliteration of dead space by suture fixation of the mastectomy flaps to the underlying chest wall, on the amount and duration of postoperative fluid drainage and incidence of seroma formation after breast surgery.

## Methods

This is a prospective comparative study that was conducted between June 2009 and July 2010 on forty patients with breast carcinoma scheduled for modified radical mastectomy. Axillary lymphadenectomy, extending from the lower border of the axillary vein superiorly, from the medial border of the pectoralis minor muscle medially, as far as the fourth intercostobrachial nerve inferiorly and to the edge of the latissimus dorsi muscle laterally.

To be enrolled in the study, patients were required to have no alterations in their blood clotting or immune systems, or at least not be receiving anticoagulant treatment; have no psychological changes; have no uncompensated diabetes or advanced liver disease; not be severely obese, and not have had previous surgery on the axillary lymphatic system or any immediate reconstructive surgery. Short-term antibiotic prophylaxis was applied.

Informed consent was obtained from all the patients of the study for surgery and publication.

The patients were randomly divided into 2 groups, the study group (20) and the control group (20).

In the study group; after completing the modified radical mastectomy procedure, using fine absorbable sutures (vicryl 3/0), multiple alternating stitches 3 cm apart were taken, in raws, between the subcutaneous tissues of the skin flaps and the underlying muscles at various parts of the flap and also, at the wound edge. Special attention is taken to the obliteration of the largest potential dead space, the empty axillary apex. Closed suction drains are used.

In the control group; after mastectomy, the wound was closed in the conventional method at the edges and closed suction drains are, also used.

Patients and tumor characteristics and operative related factors were recorded. The amount and color of drained fluid were, also, recorded daily. The drains will be removed when the amount becomes less than 50 cc, or the drained fluid started to become infected disregarding the amount drained in the last days.

Local chest wall ultrasound over the flaps and axilla will be done 2 weeks after removal of drains to document or exclude the presence of any collections. The total amount and duration of drained fluid and the formation of seroma were recorded.

The results were compared between the two groups and the effect of flap fixation on the amount and duration of fluid drainage and formation of seroma were concluded. The Mann-Whitney test was used to assess differences between the 2 patient groups.

## Results

Characteristics of patients of our study were compared between the 2 studied groups and summarized in table 1. There was no significant difference between the two studied groups concerning the age, the histological type, stage, grade and administration of neoadjuvant chemotherapy.

**Table 1:** Tumor Characteristics of the two studied groups

	Flap fixation Group (n = 20)	Control Group (n = 20)	P value
**Patient age**			
Mean (range), Y	51(37-62)	54(38-72)	
**Histological types**:			
Intraductal carcinoma (IDC)	18	18	
Intralobular carcinoma (ILC)	1	1	1.00
Mixed	1	1	
**Grade**			
II	16	15	
III	4	5	1.00
**T stage**			
T1	1	1	
T2	15	10	0.242
T3	4	6	
T4	0	3	
**N Stage**			
N0	12	6	
N1	6	10	0.178
N2	2	4	
**Neoadjuvant Chemotherapy**			
Yes	3	6	
No	17	14	0.451

Operative related features were comparable between the two groups. There was no significant differences between the two groups in volume of the tumor mass removed (p = 0.125) and area of skin removed (p = 0.829). Similarly, there was no significant difference between the two groups in the number of lymph nodes removed, either the total number (p = 0.273) or the number of positive nodes (p = 0.775) (table 2).

**Table 2:** Operative features of the two studied groups

	Flap fixation Group (n = 20)	Control Group (n = 20)	p value
**Volume of the Tumor Mass Removed:**			
**Mean (Range) (ml)**	332.8(14.1-3592.8)	465.5(10.3-2145.5)	0.125
**Area of the Skin Removed:**			
**Mean (Range) (cm^2^)**	827.7(471.4-1885.7)	821.9 (251.4-1386.0)	0.829
**Total Number of lymph nodes Removed:**			
**Mean (Range)**	18.9 (12.0-30.0)	20.8(7.0-56.0)	0.273
**Number of Positive lymph nodes removed:**			
**Mean (Range)**	2.8 (0.0-18.0)	5.9 (0.0-55.0)	0.775

Table 3 shows that in the flap fixation group, the drain was removed in significantly shorter time compared to the control group (p < 0.001), also the total amount of fluid drained was significantly lower in the flap fixation group (p < 0.001). During the last days before drain removal, the amount of drained fluid was comparable in the two groups (p = 0.175), however the amount drained in the last day was significantly lower in the flap fixation group (p = 0.002).

**Table 3:** Postoperative outcome measures

	Flap Fixation Group (n = 20)	Control Group (n = 20)	p value
**Day of Drain Removal**			< 0.001
Mean (Range)	5.0(2.0-12.0)	13.4(5.0-22.0)	

**Total amount of drained fluid (ml)**			
Mean (Range)	524.8(170.0-1525.0)	2017.8(445.0-5615.0)	< 0.001

**Amount of drained fluid in the last 3 days (ml)**			
Mean (Range)	207.8(130.0-300.0)	213.0(125.0-600.0)	0.175

**Amount of drained fluid in the last day (ml)**			
Mean(Range)	35.0(20.0-50.0)	51.5(25.0-200.0)	0.002

The flap fixation group showed a significantly lower frequency of seroma formation compared to the control group, both clinically (p = 0.028) and ultrasonographically (p = 0.047). Because ultrasonography (US) is a more accurate tool for diagnosis of seroma formation, we considered the US diagnosis as the definitive diagnosis of seroma. The complication rate was less in the flap fixation group, 2 patients (10%) developed cellulitis, while in the control group, 2 patients developed cellulitis and 2 developed partial flap necrosis (20%) (table 4).

**Table 4:** Frequency of seroma and complications in the two groups

	Flap fixation Group (n = 20)	Control Group (n = 20)	P value
**Seroma (clinical):**			
No seroma	18 (90.0%)	12 (60.0%)	
G2 seroma	2 (10.0%)	7 (35.0%)	0.028
G3 seroma	0	1 (5.0%)	
**Seroma (ultrasonographic):**			
No seroma	16 (80.0%)	10 (50.0%)	
G1 seroma	2 (10.0%)	2 (10.0%)	
G2 seroma	2 (10.0%)	7 (35.0%)	0.047
G3 seroma	0	1 (5.0%)	
**Complications**			
Cellulitis	2	2	
Partial flap necrosis	0	2	

In this study, the value of flap fixation technique is most evident in the patient with bilateral breast cancer treated by bilateral simultaneous mastectomy. The right was LOQ T2 N0 IDC operated in the conventional manner, while the left was retroareolar T2N1 IDC closed by flap fixation technique. On the left side the drain was removed after 3 days with total drainage volume of 200 cc and no complications in this side, while on the right side, the drain was removed after 13 days with total drainage volume of 1720 cc, and this side developed G2 seroma that was repeatedly aspirated and developed partial flap necrosis that was treated conservatively.

## Discussion

Axillary dissection remains an integral part of breast cancer treatment for prognostic and curative purposes. It is possible to avoid axillary dissection in selected patients (T1N0) using the sentinel lymph node technique. However, in the majority of cases, axillary lymphadenectomy is not avoidable and still has complications, in particular seroma formation (15%-81%), which can delay the patient's discharge, healing, and supplementary radiotherapy and chemotherapy treatments [1,3,8,9].

The pathogenesis of seroma has not been fully understood. Seroma is formed by acute inflammatory exudates in response to surgical trauma and acute phase of wound healing. Extensive dissection in mastectomy and axillary lymphadenectomy damages several blood vessels and lymphatics with subsequent oozing of blood and lymphatic fluid from a larger raw surface area when compared with breast-conserving procedures leads to seroma formation. Fluid accumulation elevates the flaps from the chest wall and axilla thereby hampering their adherence to the chest wall bed and delay healing [10].

Petrek et al. [11] in a prospective randomized trial showed that the most significant influencing factors in the causation of seroma were the number and the extent of axillary lymph node involvement. However, Gonzalez et al. [12] and Hashemi et al. [13] reported that the only statistically significant factor influencing the incidence of seroma formation was the type of surgery. They reported higher seroma rate in modified radical mastectomy than following wide local excision and axillary dissection due to larger dead space found after mastectomy.

Although a number of factors have been correlated with seroma formation, there was no risk factor supported by strong evidence. However there was moderate evidence (grade B) to support the increased risk of seroma formation in individuals with heavier body weight, extended radical mastectomy as compared with simple mastectomy, and a greater initial three day drainage volume and reduced seroma formation when SLNB replace axillary dissection [14].

Present evidence correlate increase in the incidence of postoperative seroma to electrocautery because of increased thermal trauma [15]. The influence of this factor was abolished in this study, as all mastectomies in both groups were performed by one surgeon using the same technique and used electrocautery in all cases.

In assessing the severity of seroma, the Common Terminology Criteria for Adverse Events v3.0 grades seroma as; grade 1 if asymptomatic, grade 2 if symptomatic (medical intervention or simple aspiration indicated), and grade 3 if symptomatic (interventional radiology or operative intervention indicated). According to this definition, the majority of seromas documented would be categorized as grade 2, and as grade 3 in rare cases; however, grade 1 have so far been underestimated [6].

In terms of the methods of reducing seroma magnitude, there have been numerous reports of conflicting results in literature of the benefits of using an external compression dressing [16], immobilization of the arm [17], the use of fibrin glue [18], excessive use of the electric scalpel compared to ligature of the lymphatic branches [19], benefits of multiple drains, and the type of suction (high or low pressure) applied [20,21].

Several preliminary or retrospective studies, and prospective studies, as well as RCTs have found it useful to close the dead space by securing the flaps to the chest wall with sutures [22-27]. Larsen *et al. *[23] reported that this method allowed smoother, more prompt recovery, and less disability after mastectomy. Similarly, this technique significantly reduced the incidence of seroma formation, breakdown of wound edges, and prolonged serous discharge, and did not lead to a reduced functional range of shoulder motion among patients who underwent conventional mastectomy. Moreover, it allowed the early and safe removal of drains at 48 hours or 72 hours after surgery [22,26].

It is interesting to note that in the RCT by Purushotham *et al. *[28], breast surgery without drainage did not increase surgical or psychological morbidity including seroma formation if flaps were fixed with sutures, and early discharge as a consequence of avoiding wound drainage resulted in an overall reduction in cost. Similarly, the axillary flap fixation with sutures was useful to avoid axillary drainage in patients undergoing BCS and conventional axillary lymph node dissection [29].

In our study; the clinical incidence of seroma was 25% (10/40), most of them are grade 2 (90%). Four more cases were detected by ultrasonography, increasing the overall incidence to 35% (14/40), the 4 cases were grade 1 minor seromas that are clinically asymptomatic and not detected by the patient. This is the first study to use chest wall ultrasonography for diagnosis of seroma. Our overall incidence of seroma falls within the range of incidence reported by most authors that varies widely between 15 and 81% [1].

By comparing the results of the two groups of our study, we found that; flap fixation technique is associated with a lower incidence of seroma (10%) after mastectomy as compared to the control group (40%) with P-value = 0.028. Several investigators have also found that flap fixation technique is useful in decreasing seroma formation [22,23,28].

Currently, there is more concern over the incidence of seroma, than the more important quantity of serum lost and duration of drainage. Although not always measured, drainage volumes can exceed 2 L, and volumes up to 3.7-5 L are on record. More than 30 aspirations over periods of 2-3 months have been reported [30]. Published reports focused on studying the effect of obliteration of the dead space on seroma formation but, did not address the effect on the amount of drained fluid and drainage period. Up to our knowledge; studying this relation in our work had never been mentioned in literature before.

In this study, we found that; the flap fixation technique significantly decreases the total amount of fluid drained with mean drainage volume of 524 c.c. in the flap fixation group versus 2017 in the control group (P value < 0.001). It has been reported by some authors that the total drainage volume may reach up to 5 liters if the technique of flap fixation was not used [31].

We found that, this technique significantly decrease the drainage period. The mean duration of drainage is 5 days in the flap fixation group versus 13.4 days in the control group (P < 0.001). Most surgeons tend to remove the drain when the drainage volume is less than 50 mL in the preceding 24 h and this usually take up to 10 days [32].

In our series, the overall complications rate is 15% (6/40) of cases with no mortality. This rate is less than that reported in most studies. Reported studies document that surgical morbidity from breast and/or axillary wound occur in up to 30% of cases [33]. Additionally, in the group with flap fixation only 2 cases (10%) developed cellulitis that was treated medically, while in the control group 2 cases developed cellulitis and 2 cases developed partial flap necrosis, this mean that the morbidity is less with flap fixation.

## Conclusion

The findings of the literature review and based on our experience, it is suggested that the flap fixation technique is a valuable procedure that significantly decrease the total amount of drained fluid, allowing the earlier removal of the drains as well as decreasing the incidence of seroma formation, and the need for frequent visits for seroma fluid aspiration after mastectomy. However, this technique should be tried on a much wider scale to prove its validity in decreasing the incidence of seroma formation and its subsequent complications, so that it can be introduced as a step in the mastectomy operations.

## List of abbreviations

BCS: Breast Conserving Surgery; IDC: Invasive Duct Carcinoma; LOQ: Lower Outer Quadrant; RCTs: Randomized Controlled Trails; SLNB: Sentinal Lymph Node Biopsy; US: Ultrasonography.

## Competing interests

The authors declare that they have no competing interests.

## Authors' contributions

The author conceived and design the study, performed surgery. The author, also write the manuscript and approved it after final revision.

## Authors' informations

Lecturer of surgical oncology, Surgery Department, National Cancer Institute, Cairo University, Cairo, Egypt
